# Chemical Imaging of MXenes with X‑ray Microscopy

**DOI:** 10.1021/acs.accounts.6c00324

**Published:** 2026-09-15

**Authors:** Namrata Sharma, Louis Godeffroy, Tristan Petit

**Affiliations:** † Nanoscale Solid−Liquid Interfaces, 28340 Helmholtz-Zentrum Berlin für Materialien und Energy GmbH, Albert-Einstein Str. 15, 12489 Berlin, Germany; ‡ Faculty of Mathematics and Natural Sciences, TU-Berlin, Hardenbergstraße 36, 10623 Berlin, Germany; § Laboratoire de Physique de l’École normale supérieure, ENS, Université PSL, CNRS, Sorbonne Université, Université Paris Cité, 75005 Paris, France; ∥ Institut de Science des Matériaux de Mulhouse (IS2M), CNRS UMR 7361, Université de Haute-Alsace, 68057 Mulhouse, France

## Abstract

Chemical imaging combines microscopy with spectroscopy to achieve a chemical contrast, enabling the identification of different chemical species with high spatial resolution. This capability is particularly valuable for the characterization of composite and heterogeneous materials, as well as for probing chemical reactions that are spatially segregated at the micro- or nanoscale. Representative examples include catalytic processes that locally modify the state of a catalyst, or battery particles that charge nonuniformly during cycling.

Chemical imaging can be implemented using scanning probe, optical, electron, or X-ray spectromicroscopy techniques, each providing distinct forms of chemical contrast. Scanning probe and optical methods primarily probe valence electrons or vibrational modes, offering high chemical sensitivity, whereas electron and X-ray techniques typically probe core-level excitations, providing element-specific information. The latter also enable higher spatial resolution, as electrons and X-rays have much shorter wavelengths than visible light (typically <10 nm versus ∼400 nm).

Among these approaches, X-ray-based methods are particularly powerful for direct, label-free chemical imaging due to their high elemental and site specificity. They are also significantly less invasive than electron-beam techniques. The soft X-ray range (ca. 100–2000 eV) is especially relevant, as it enables the selective excitation of the K-edge of light elements such as carbon, oxygen, and nitrogen, as well as the L- or M-edge of transition metals. Despite these relatively low photon energies, spatial resolutions down to ∼10 nm can be routinely achieved using standard X-ray optics.Moreover, the information depth can be tuned through the detection mode. For instance, photoemission-based techniques such as X-ray photoemission electron microscopy (X-PEEM) provide high surface sensitivity, whereas transmission-based methods such as scanning transmission X-ray microscopy (STXM) probe the bulk of the material that is though limited to thin samples. Finally, the comparatively large penetration depth of X-rays, relative to electrons, enables the use of *in situ* cells to investigate chemical processes under realistic conditions.

In this Account, we present recent advances in X-ray chemical imaging, highlighting our work on two-dimensional transition-metal carbides known as MXenes. These materials are particularly well-suited for such approaches due to their rich surface chemistry, flat geometry, and micrometer-sized flakes. MXenes have attracted growing interest across a wide range of applications, including electromagnetic interference shielding, sensing, and energy storage. They adopt a M_n+1_X_n_T_*x*_ layered structure, where M is a transition metal, X is carbon and/or nitrogen, and T_*x*_ denotes mixed surface terminations, which can be selectively imaged by (soft) X-ray techniques. This account focuses mainly on Ti_3_C_2_T_*x*_ characterization because it is the most studied MXene to date.

Leveraging the elemental sensitivity of soft X-rays, we imaged both confined water and the MXene framework itself, in vacuum and in various aqueous environments. We further present recent results on the spontaneous and electrochemically driven intercalation of protons and alkali cations in Ti_3_C_2_T_*x*_, investigated by *in situ* STXM. These studies reveal that changes in the metal oxidation state strongly depend on the nature of the intercalant and highlight pronounced subflake variations in surface chemistry. This new chemical picture completely upended the conventional understanding of MXene chemistry, offering new keys to address challenges in their implementation for energy devices. Finally, we discuss recent developments in the field and outline promising directions for future research.

## Graphical abstract

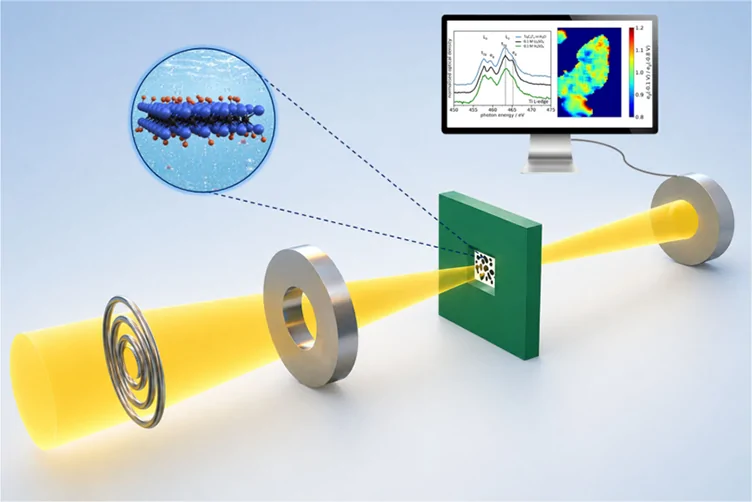

## 1 KEY REFERENCES

-

Sharma, N. ; Godeffroy, L. ; Bärmann, P. ; Amargianou, F. ; Weisser, A. ; Dessoliers, Z. ; Lounasvuori, M. ; Weigand, M. ; Petit, T. Nanoscale Chemical Imaging of Pseudocapacitive Charge Storage in MXenes. Energy Environ. Sci. 2026, 19, 2, 680–690, DOI: 10.1039/D5EE05809K PMC1276776841497339.^1^ *In this work, in situ chemical imaging of MXene flakes demonstrates changes of the Ti oxidation state upon intercalation of protons and Li* ^*+*^ *cations in aqueous electrolyte under applied potential using in situ electrochemical STXM*.
-

Amargianou, F. ; Bärmann, P. ; Sharma, N. ; Lounasvuori, M. ; Furchner, A. ; Kenaz, R. ; Ghosh, S. ; Förster, J. D. ; Pöhlker, C. ; Weigand, M. ; Petit, T. Water-Induced Local Redox Reactions on Individual Ti_3_C_2_T_x_ MXene Flakes in Aqueous Environment. Angew. Chem., Int. Ed. 2026, 65, 4, e20508, DOI: 10.1002/anie.202520508 PMC1282846441346054.^2^ *In this work, different water phases are identified in MXene flakes. Local chemical changes of MXenes upon intercalation of water and alkali cations are also demonstrated*.
-

Zhang, T. ; Mazzio, K. A. ; Wang, R. J. ; Lounasvuori, M. ; Al-Temimy, A. ; Amargianou, F. ; Mawass, M.-A. ; Kronast, F. ; Tobbens, D. M. ; Lips, K. ; Petit, T. ; Gogotsi, Y. Conductivity Hysteresis in MXene Driven by Structural Dynamics of Nanoconfined Water. Nat. Commun. 2025, 16, 1, 7447, DOI: 10.1038/s41467-025-62892-7 PMC1234393640796749.^3^ *In this work, a phase transition of confined water at low temperature is identified by X-ray chemical imaging using X-PEEM based on changes of the water electronic signature*.
-

Amargianou, F. ; Bärmann, P. ; Shao, H. ; Taberna, P. ; Simon, P. ; Gonzalez-Julian, J. ; Weigand, M. ; Petit, T. Nanoscale Surface and Bulk Electronic Properties of Ti_3_C_2_T_x_ MXene Unraveled by Multimodal X-Ray Spectromicroscopy. Small Methods 2024, 8, 12, 1–9, DOI: 10.1002/smtd.202400190 PMC1167185538874117.^4^ *In this work, the characterization of MXene flakes with bulk and surface sensitivity using transmission and electron yield detection, respectively, is demonstrated with Scanning X-ray Microscopy*.

## 2 Introduction

Chemical imaging is a powerful methodology to track local chemical processes, as it combines microscopy with spectroscopy to achieve chemical contrast. Depending on the microscopy technique, the accessible spatial resolution spans from the millimeter to the nanometer scale. In the context of energy materials, however, where nanomaterials or nanostructured materials are ubiquitous, nanoscale resolution is often required, and significant efforts have recently been devoted to developing chemical imaging approaches at this scale. Among the available techniques, including scanning probe,^5–9^ optical,^10^ electron,^11^ mass (ToF-SIMS),^12^ and X-ray microscopies,^13–15^ the latter stand out for their sub-50 nm spatial resolution and intrinsic, label-free chemical contrast. In particular, soft X-ray spectromicroscopy is highly sensitive to transition metal oxidation states, making it especially relevant for battery science and catalysis, where activity is often governed by transition metal centers. Its applicability has been further expanded by recent advances in *in situ* cell design, enabling imaging in gas and liquid environments,^2,16,17^ as well as under electrochemical control.^1,18–20^

The pioneering work of Frank de Groot and co-workers in 2008, which demonstrated chemical imaging of a working catalyst using scanning transmission X-ray microscopy (STXM) for the first time, marked the starting point for the development of *in situ* STXM.^16^ By adapting a nanoreactor initially designed for *in situ* transmission electron microscopy (TEM), they showed that STXM could operate at atmospheric pressure and temperatures up to 350 °C, enabling the monitoring of phase changes in a complex iron-based Fischer–Tropsch catalyst, as well as the nature and spatial distribution of the carbon species formed, representing a significant breakthrough. Beyond the Fischer–Tropsch reaction, which remains an active area of research,^17^ this work stimulated numerous studies of other thermocatalytic processes, including dry methane reforming^21^ and CO oxidation,^22^ often with only minor modifications to the original cell design.

While integrating a heating element at these scales was a major challenge at the time, precise control over the distance between the two SiN_*x*_ windows enclosing the cell was comparatively less critical. Although the penetration depth of soft X-rays is limited, transmission through several micrometers of gas at atmospheric pressure results in only modest attenuation: for example, 10 μm of air at 1 bar attenuates the incident beam by only ∼ 2% at the O K-edge (530 eV).^23^ In contrast, the same thickness of a liquid phase (e.g., water) leads to nearly complete absorption at this energy, which significantly hindered the development of liquid-phase STXM for several years.

Driven by the large *in situ* TEM community, which pushed the development of liquid cells with well-controlled thickness, an even more critical parameter given the shorter probing depth of electron beams, the first liquid-phase STXM measurements, including under electrochemical control, were reported by William Chueh and co-workers in 2016.^18^ In their effort to elucidate Li^+^ intercalation pathways in Li_*x*_FePO_4_ (LFP), a model Li-ion battery cathode material, they had previously performed *in situ* fluorescence yield X-ray microscopy (FY-XRM), where thickness constraints are relaxed since the emitted photons are collected through the same window.^24^ Despite the lower spatial resolution, these earlier measurements revealed that ellipsoidal particles intercalate sequentially, whereas platelet-shaped particles intercalate more uniformly, likely reflecting differences in surface properties arising from synthesis.

Using *in situ* STXM, they were subsequently able to resolve these mechanisms at the subparticle scale and demonstrated that, in platelet particles, the compositionally nonuniform solid-solution domains observed during (de)­lithiation originate from spatial heterogeneities in surface reaction rates. As LFP serves as a good model system for Li^+^ intercalation, this work was followed by numerous studies from the groups of Jongwoo Lim, Martin Z. Bazant, and William Chueh, which further extended *operando* conditions—particularly toward higher C-rates, to investigate phenomena such as particle cracking, a key degradation pathway in practical batteries.^25–28^

In the context of soft X-ray spectromicroscopy, the choice of LFP is far from arbitrary, as the Li content (x), or state of charge (SOC), is directly proportional to the average oxidation state of Fe, which can be measured with high precision. In addition, LFP can operate in aqueous electrolytes, providing greater flexibility for electrolyte handling at experimental stations that are typically not maintained under strictly controlled or dry atmospheres.

Beyond cathode materials, *in situ* STXM has also been employed to investigate processes such as Zn dendrite formation on Zn anodes in Zn-ion batteries.^29^ However, it was not until 2021 that the technique was extended more broadly beyond battery systems: in combination with complementary microscopies, William Chueh and co-workers used it to elucidate the oxygen evolution reaction mechanism on a Co­(OH)_2_ electrocatalyst.^30^ Their results revealed a progressive yet spatially nonuniform transition from Co^2+^ to Co^3+^, the latter being the active state for the reaction.

Concurrently, the introduction of ptychographic approaches to STXM significantly improved spatial resolution, reaching sub-10 nm.^31,32^ This advance opened new opportunities for *in situ* studies in electrocatalysis, where active particles are typically tens of nanometers in size or smaller. In this context, spectro-ptychography was successfully applied by Adam Hitchcock and co-workers in 2023 to track the oxidation state of Cu in sub-100 nm Cu electrocatalysts under electrochemical CO_2_ reduction conditions.^33^

As highlighted above, 2D materials (e.g., LFP and Co­(OH)_2_ platelets) are ideal systems for nanoscale chemical imaging, as they often consist of micrometer-sized, thin flakes with potentially heterogeneous surface chemistry.^34^ Often, they also exhibit distinct activities at their edges and basal planes,^35,30^ or whether they are single- or few-layered.

In the context of energy storage, 2D transition metal carbides and nitrides—so-called MXenes—have emerged as promising alternatives to conventional transition metal–based electrode materials.^36^ Owing to their layered structure, with interlayer spacings on the order of ∼ 1 nm that facilitate ion transport, they are particularly attractive for fast-charging applications. Their tunable surface chemistry further provides exceptional versatility, enabling them to host a wide range of ions in both aqueous and organic electrolytes.

However, because MXenes are typically synthesized via top-down chemical etching, their surface chemistry remains poorly defined. It comprises a mixture of functional groups (e.g., – O, – OH, and – F in Ti_3_C_2_T_*x*_ produced by conventional HF etching), which may be spatially heterogeneous, evolve over time, and differently influence the reactivity of the underlying metal centers.^37^ This complexity makes it extremely challenging to establish clear composition–structure–property relationships governing charge storage and stability.^38^ In this context, *in situ* X-ray chemical imaging offers a powerful and promising approach to probe these relationships and, more broadly, the chemistry of MXenes.

In this Account, we first outline the basic principles of X-ray microscopy for chemical imaging. We then provide a comprehensive overview of our own research with a focus on the chemical imaging of a prototypical MXene, Ti_3_C_2_T_*x*_ MXene. In particular, we summarize recent results on probing intercalated water within MXene layers and on the evolution of the metal bonding environment upon alkali-ion intercalation, whether spontaneous or under an applied potential. Challenges and opportunities associated with characterizing MXenes with soft X-ray microscopy are highlighted. Finally, we discuss future directions and perspectives for the field.

## 3 CHEMICAL IMAGING WITH X-RAY MICROSCOPY

X-ray microscopy is a powerful set of techniques that enables the visualization and analysis of materials with high spatial resolution, combining structural and chemical information. By exploiting the interaction of X-rays with matter, X-ray microscopy enables element-specific and state-specific analysis, allowing selective investigation of elements in MXenes such as Ti, V, Mo and Nb on the transition metal side, and C, N, O and F on the core and surface side.^39–42^ Briefly, when X-rays interact with the sample, the core state electrons are excited either into the unoccupied valence states or the continuum via the photoelectric effect.^43,44^

If the electron ends up in a valence state, it can relax radiatively by going back to the core hole and emitting an X-ray photon (fluorescence) or nonradiatively through processes such as Auger decay as schematized in Figure 1. The absorption spectrum can be recorded by measuring X-ray fluorescence photons, emitted electrons, or in transmission mode. In transmission mode, the X-rays transmitted through the thin sample are measured, and the absorption coefficient is determined using the Beer–Lambert law, providing a direct measurement of bulk absorption.^45^

**Figure 1:**
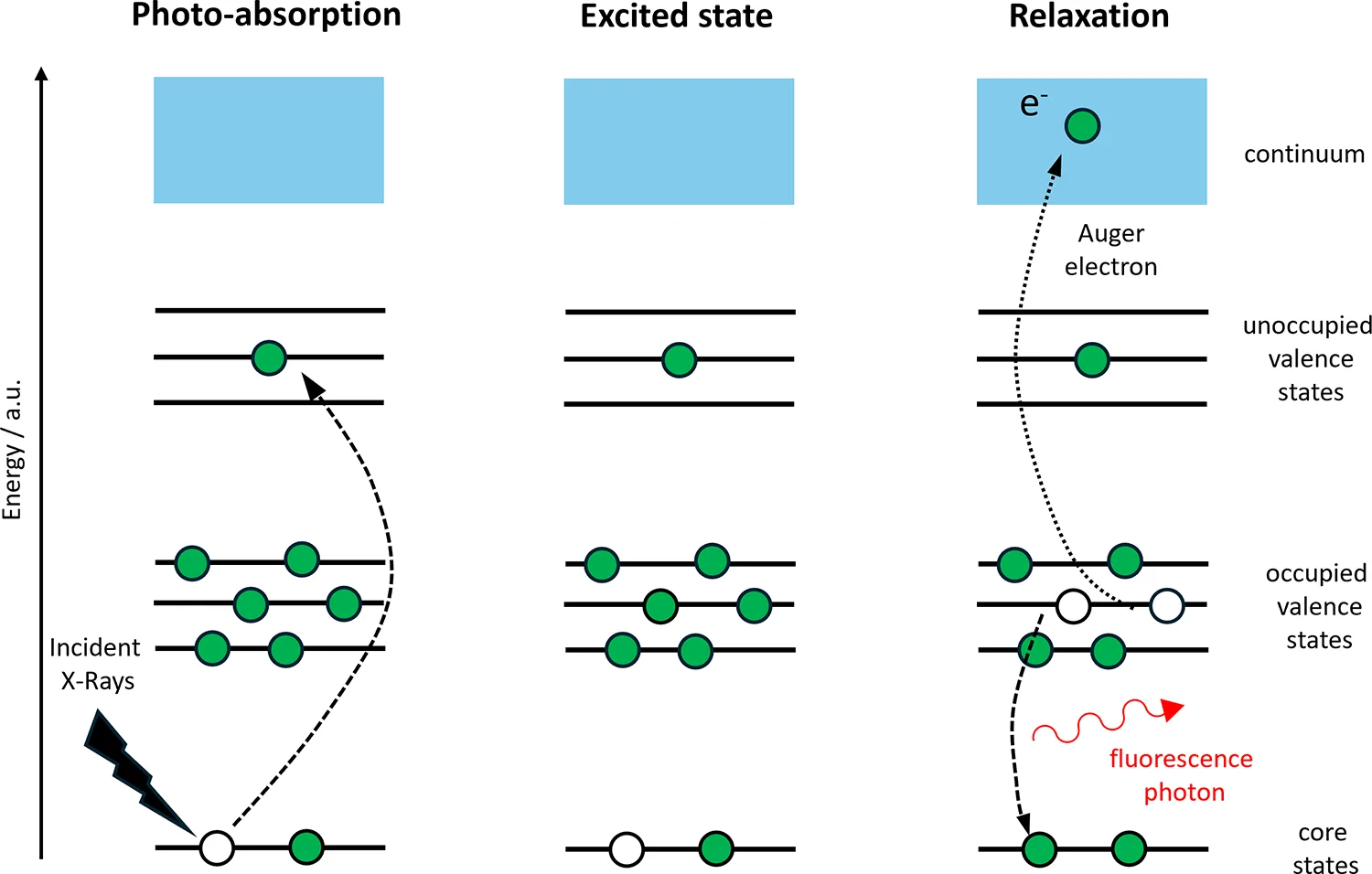
**Schematic of the photoabsorption and relaxation processes occurring in X-ray absorption spectroscopy (XAS).** An incident photon, whose energy corresponds to an XAS resonance, excites an electron from a core level into an unoccupied valence state. This excited state is unstable, and the atom relaxes to emit either a fluorescent photon or eject an Auger electron. Reproduced with permission from ref.^46^ Copyright 2006 Elsevier.

Soft X-rays, in the energy range of approximately 100 eV to 2 keV, are well suited for probing light elements and surface-related chemical states, whereas hard X-rays above 2 keV have greater penetration depth and are used to study heavier elements. For example, the K-shell electrons of carbon and oxygen can be excited using soft X-rays, while elements with higher atomic numbers, such as titanium or vanadium, exhibit higher binding energies that require hard X-ray energies to access their K-shell electrons, although their L-shell electrons can be accessed with soft X-rays.^47,48^ Both the K-edge and L-edge of Ti or V provide valuable information on variations in their oxidation states and local coordination environments at the molecular scale. While Ti K-edge XAS is most employed in research on Ti_3_C_2_T_*x*_ MXenes, Ti L-edge spectroscopy offers stronger transition intensities, providing enhanced sensitivity to the octahedral and distorted local symmetries present in MXenes with diverse surface terminations. This makes soft X-rays particularly well-suited for studying MXenes.

X-ray spectroscopy, combined with microscopy, provides spatially resolved information about the distribution of different elements and their chemical states within a single flake. Different X-ray microscopy techniques offer complementary insights depending on the sample and the information sought. X-ray Photoemission Electron Microscopy (XPEEM) is surface-sensitive, probing the top few nanometers and providing chemical-state maps through photoelectron detection. Transmission X-ray Microscopy (TXM) images the bulk of thicker samples using transmitted X-rays and can be combined with X-ray absorption contrast to provide 2D or 3D chemical mapping.^49^ Scanning Transmission X-ray Microscopy (STXM) combines high-resolution imaging with XAS, providing pixel-by-pixel chemical mapping of thin samples with high spatial and chemical sensitivity.^50^ While STXM is essentially based on transmission measurements, secondary processes also allow imaging using electron yield^4^ or fluorescence yield.^51^ Together, these techniques enable comprehensive analysis from surface to bulk, linking morphology, elemental composition, and chemical state.

STXM measurements can generally be performed in three spatial modes: point scans corresponding to a full energy scan at one position, line scans corresponding to an energy scan along a line or image scans corresponding to a sequence of images at different energies as shown in Schematic of Figure 2. The latter is commonly referred to as hyperspectral imaging, where each pixel is constituted of a spectrum. In a point scan, the X-ray beam is focused on a single selected position of the sample, and the absorption signal is recorded as a function of photon energy, providing detailed local spectroscopic information (Figure 2a). In contrast, hyperspectral imaging involves acquiring a series of images over a range of photon energies, producing an energy-resolved image sequence (Figure 2b). Hyperspectral imaging allows both spatial and chemical information to be extracted, enabling the mapping of elemental composition and chemical states across the sample with high spatial resolution.

**Figure 2:**
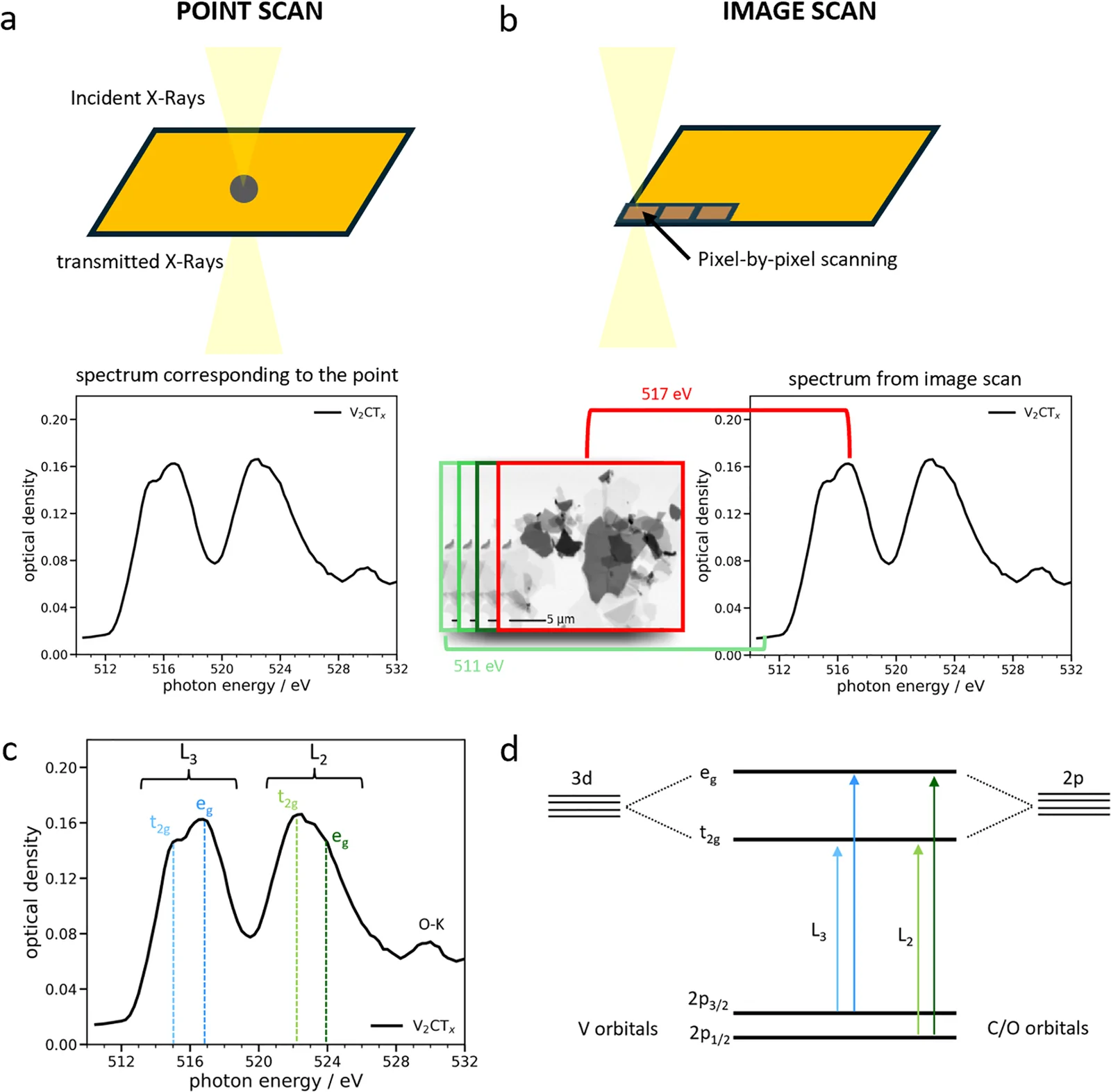
**X-ray absorption spectroscopy of V**_**2**_ **CT_*x*_ MXenes.** Schematic of a) point scan and b) image scan acquisition in STXM. c) XAS spectra of V L-edge and O K-edge of V_2_CT_*x*_ MXenes in vacuum, d) simplified schematic of orbital splitting expected for V in V_2_CT_*x*_ MXenes.

It has to be noted that the interaction of highly focused X-ray beams with matter, at the core of X-ray microscopy, may induce radiation damage to the investigated sample. The X-ray dose has therefore to be reduced as much as possible. To minimize radiation damage, hyperspectral imaging measurements are preferred over point (and line) scans since the measurement time per pixel is significantly reduced (ca. 1 vs 1000 ms). To further minimize beam effects and measurement time, energy-selective imaging can be done at selected energies instead of acquiring full spectra with a constant energy step. Additionally, for *in situ* studies in water, the generation of radiolytic species may induce local chemical reactions with the sample, which can be limited by ensuring a constant liquid flow during the measurement. For *in situ* electrochemical experiments, control experiments that compare the chemical changes induced by X-rays in the absence of an applied potential with those observed under actual experimental conditions also help assess the extent of beam damage.

To monitor the oxidation state changes of transition metals such as Ti and V, the variation in the L_3_ and/or L_2_ e_g_/t_2g_ and/or L_3_/L_2_ peak intensity ratios are analyzed. The V (and Ti) L-edge corresponds to the electronic transitions of the 2p_3/2_ and 2p_1/2_ core electrons to the 3d valence orbitals, resulting in characteristic L_3_ and L_2_ edges, respectively. In MXenes such as Ti_3_C_2_T_*x*_, Ti_2_CT_*x*_, and V_2_CT_*x*_ MXenes, the transition metal atoms are coordinated in an octahedral environment with carbon and oxygen atoms. The electric field from the ligands (C/O) interacts with the 3d orbital of the transition metal, resulting in their splitting into t_2g_ (d_*xy*_, d_*xz*_, d_*yz*_) and e_g_ (d_z^2^_, d_x^2^–y^2^_) symmetry orbitals (crystal field splitting, Figure 2c&d).^49,41^ The relative peak intensity of these states reflects the density of unoccupied d-states of the transition metal atoms and is therefore directly related to their oxidation state (loss of electron → unoccupancy of 3d orbital increases → e_g_ peak intensity increases→L_3,2_ e_g_/t_2g_ peak intensity increases). Hence, an increasing L_3,2_ e_g_/t_2g_ peak intensity ratio suggests a higher oxidation state of V or Ti.^4^

The O K-edge also exhibits two distinct peaks at approximately 529 and 530.5 eV corresponding to the O 2p orbitals hybridized with the Ti/V 3d orbitals with t_2g_ and e_g_ symmetries, respectively. Hence, similar information regarding the metal oxidation state can be deduced from the O K-edge as well. However, the detected signal is weaker than the metal L-edge and we found it difficult to image *in situ* in liquid the signature of MXene surface chemistry at the O K-edge. Similarly, while chemical imaging at the C K-edge would allow the probing of the carbide core of MXenes, which was found to be sensitive to surface chemistry,^42^ our attempts were often limited by the signal-to-noise ratio obtained for few-layered MXenes.

## 4 Chemical Imaging of Individual Mxene Flakes

First measurements on individual MXene flakes were performed by XPEEM in vacuum on Ti_3_C_2_T_*x*_ MXenes, which has the advantage for our initial attempts of not being limited by the thickness of the MXene flakes.^52^ We have evidenced an oxidation of the MXene flake edges by monitoring the oxygen K-edge. Upon intercalation with urea^52^ and alkali ions,^53^ a more uniform oxidation of the flake was evidenced. These conclusions were, however, limited to the top MXene layer due to the limited information depth of XPEEM. We have therefore continued our efforts in implementing STXM for the characterization of individual MXene flakes.^4^ As explained above, STXM is bulk sensitive and requires, therefore, delaminated MXene flakes of fewer than ∼ 40 layers. In this regime, the quantitative extraction of the MXene thickness is possible from the measured optical density. As shown in Figure 3a, MXene flakes with a thickness between 1 and 5 layers are identified based on their optical density. No clear change of the XAS at the Ti L-edge could be identified depending on the thickness (Figure 3b), showing that these MXenes have a relatively homogeneous surface chemistry from flake to flake. In addition, the simultaneous detection of transmission and electron yield signals enables the monitoring of both surface and bulk components,^4^ thereby allowing a direct comparison with XPEEM characterization. No thickness-dependent information can be extracted from the electron yield but the contrast is rather governed by the electron emission properties, affected by the contact to the substrate, for example.

**Figure 3:**
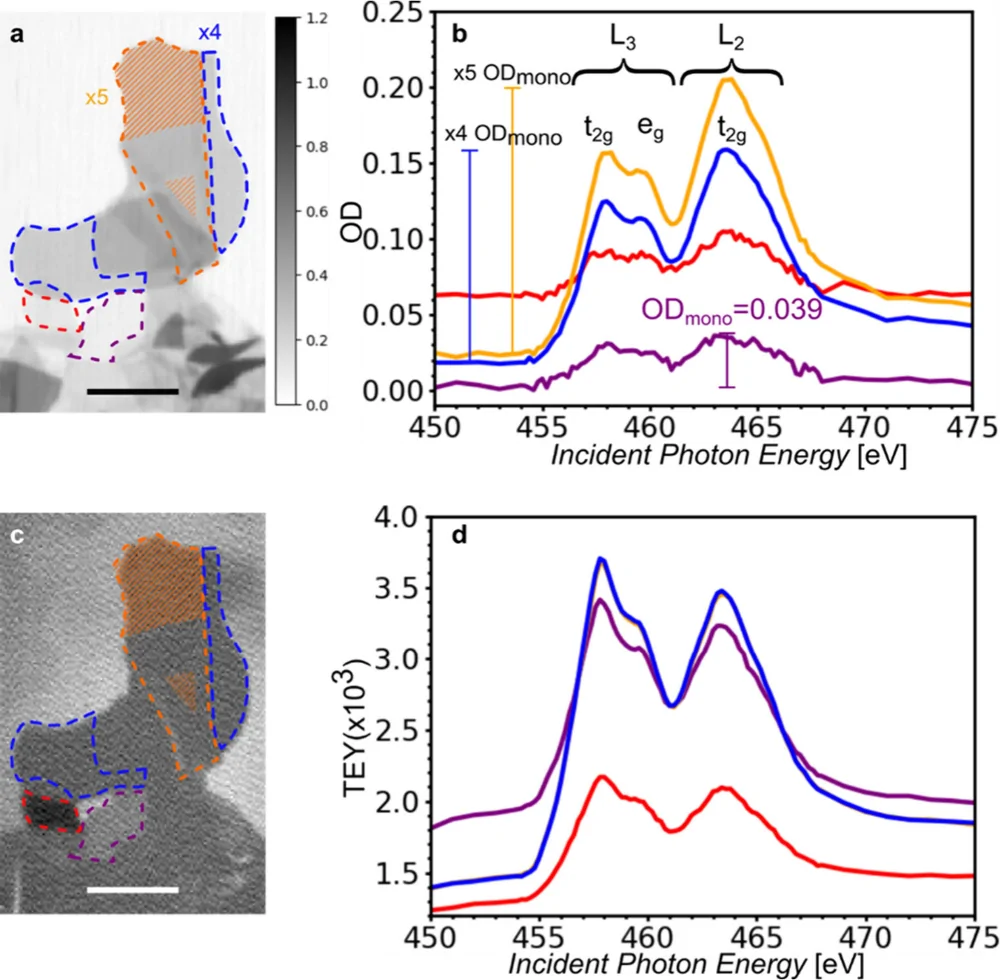
**STXM imaging of individual few-layered Ti**_**3**_ **C**_**2**_ **T_*x*_ MXene flakes.** a) Transmission image, averaged over the Ti L-edge, highlighting regions corresponding to monolayer (dotted violet), 4-layered (dotted blue) and 5-layered Ti_3_C_2_T_*x*_ MXene flakes, with corresponding transmission spectra extracted from the dashed areas (b). A monolayer MXene flake with reduced electron yield (dotted red) is also indicated. c) TEY image at 450 eV depicting the same regions as in (a), with corresponding TEY spectra (d). Scale bar: 2 μm. Reproduced with permission from reference.^4^ Copyright 2024 Wiley.

It must be noted that no edge oxidation was detected on these freshly HF-etched Ti_3_C_2_T_*x*_ MXenes (Figure 3), therefore the oxidation observed in early XPEEM measurements was most likely due to the aging of the delaminated MXene flakes stored in water. This was later confirmed by imaging MXene flakes stored for a few months in water (Figure 4).^2^ Chemical imaging at the oxygen K edge highlights an increasing oxygen content at the edges (Figure 4a,c,f), which is correlated with an increased Ti oxidation state as imaged at the Ti L-edge (Figure 4b). Transmission electron microscopy on similarly synthesized Ti_3_C_2_T_*x*_ MXenes have shown some titanium oxide nanoparticles formed between the layers (Figure 4d) and defective edges (Figure 4e), which are prone to oxidation. The high chemical sensitivity of STXM is therefore enabling the mapping of oxidized areas at the subflake level in vacuum, which is visible by monitoring both the Ti L-edge and the O K-edge.

**Figure 4:**
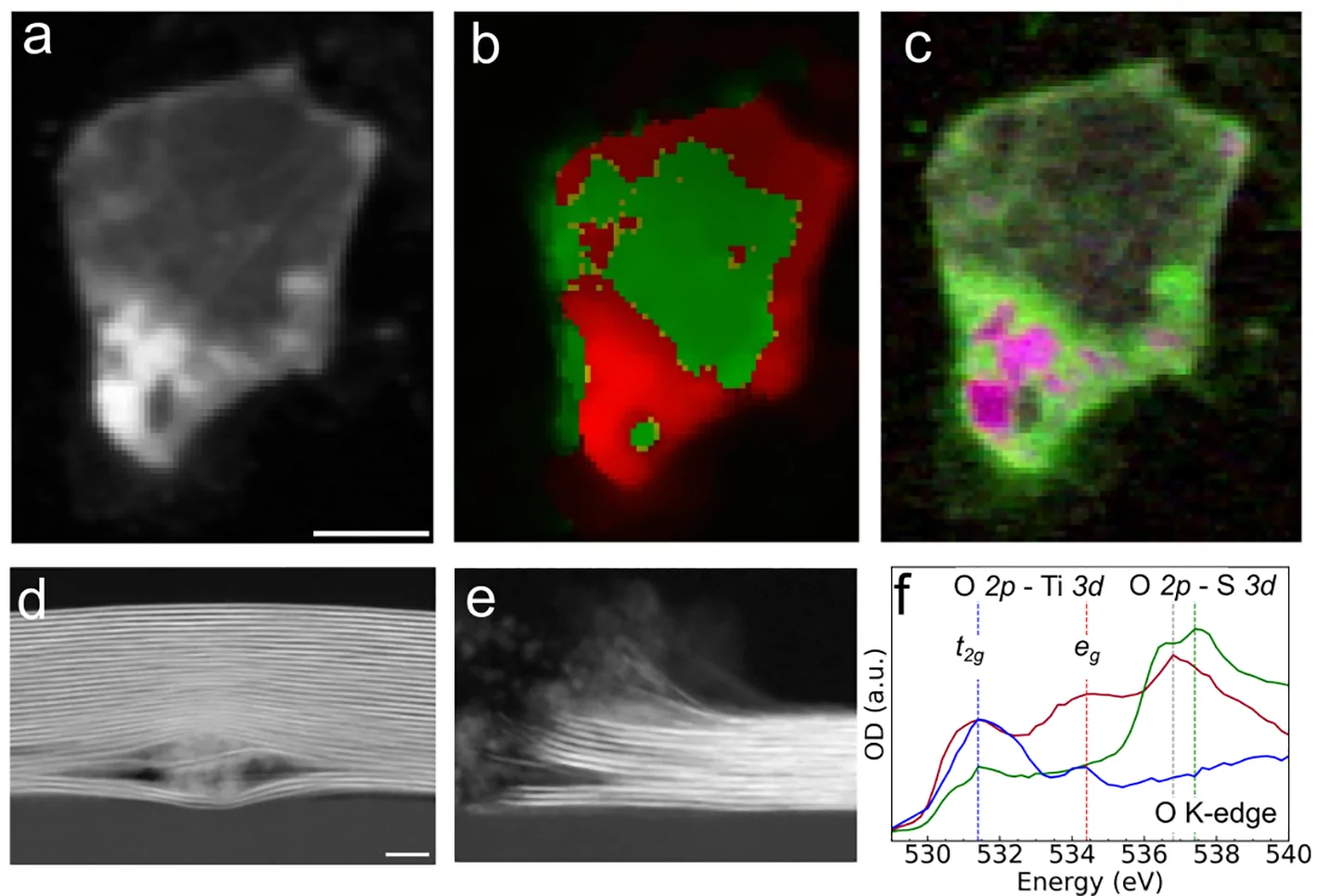
**Chemical imaging of an Ti**_**3**_ **C**_**2**_ **T_*x*_ MXene flakes after long-term storage in water.** The flake was exposed to 0.1 M H_2_SO_4_ before measurement in vacuum. a) STXM image of the flake at the oxygen K-edge. Scale bar: 2 μm. b) STXM image at the Ti L-edge color-coded based on the Ti L_2_ e_g_/t_2g_ peak intensity ratio: > 0.98 (red) and <0.98 (green). c) Chemical mapping created by STXM images at 531.4 (blue), 534.4 (red) and 537.4 (green) eV. Cross-sectional STEM images of TiO_2_ nanoparticles trapped d) between Ti_3_C_2_T_*x*_ MXene layers, and e) at the edge of a MXene flake. Scale bar: 10 nm. f) XAS spectra at the O K-edge of pristine MXene (blue), oxidized MXene (red) and MXene exposed to H_2_SO_4_ (green). Reproduced with permission from reference.^2^ Copyright 2026 Wiley.

While the high chemical sensitivity is clearly a strength of X-ray imaging, it creates constraints for sample preparation. Indeed, MXene samples must be synthesized shortly before the beamtime to avoid signatures of spontaneous oxidation. This is most particularly true when characterizing less stable MXenes such as Ti_2_CT_*x*_ which we recently imaged and found particularly sensitive to oxidation in air.^54^ Furthermore, delamination of the MXene flakes is necessary for STXM characterization, as multilayered MXene are absorbing too much for reliable imaging in transmission.^4,54^ This explains why most of our current work remains limited to Ti_3_C_2_T_*x*_ MXenes. However, we have recently demonstrated that Ti_2_CT_*x*_ ^54^ and Mo_2_CT_*x*_ MXenes^55,56^ can also be characterized by STXM at the Ti L- and Mo M-edge, respectively. We have also successfully characterized V_2_CT_*x*_ at the V L-edge and Nb_2_CT_*x*_ at the Nb L-edge, which shows that the element-sensitivity of STXM is particularly well-adapted to the rich family of MXene materials.

## 5 Chemical Imaging of Intercalated Water

The element selectivity of X-ray spectroscopy allows the monitoring of intercalated water molecules and of oxygenated surface functional groups at the O K-edge. The chemical shift induced by the different bonding environments of oxygen in water molecules or on the MXene surface allows the selective monitoring of the water molecules, which have been characterized in different conditions.^2,3^ The XAS at the oxygen K-edge of water molecules is also highly sensitive to the hydrogen bonding environment of confined water molecules.

In Zhang et al, we have monitored the freezing of intercalated water at low temperature using *in situ* XPEEM (Figure 5a).^3^ Interestingly, the chemical imaging revealed two different bonding environments, which are attributed to ice clusters with sizes of a few hundred nanometers, with a strong component at 540 eV, and to 2D water film confined in the MXene interlayer with a sharp peak at 537 eV instead (Figure 5b,c). The water electronic response is constituted of three components corresponding to a pre-, main- and postedge that can be used for chemical imaging with three different colors in a similar manner to a RGB camera (Figure 5f). Upon increasing the temperature above 260 K, a progressive melting of the ice clusters is evidenced, as well as a restructuring of water hydrogen bonding in the 2D water film with the formation of stronger hydrogen bonds (Figure 5d-f). This is visible by the progressive increase of the main-edge at 537 eV. The confined water layer is fully removed upon annealing at 590 K in ultrahigh vacuum and this component is not visible anymore.

**Figure 5:**
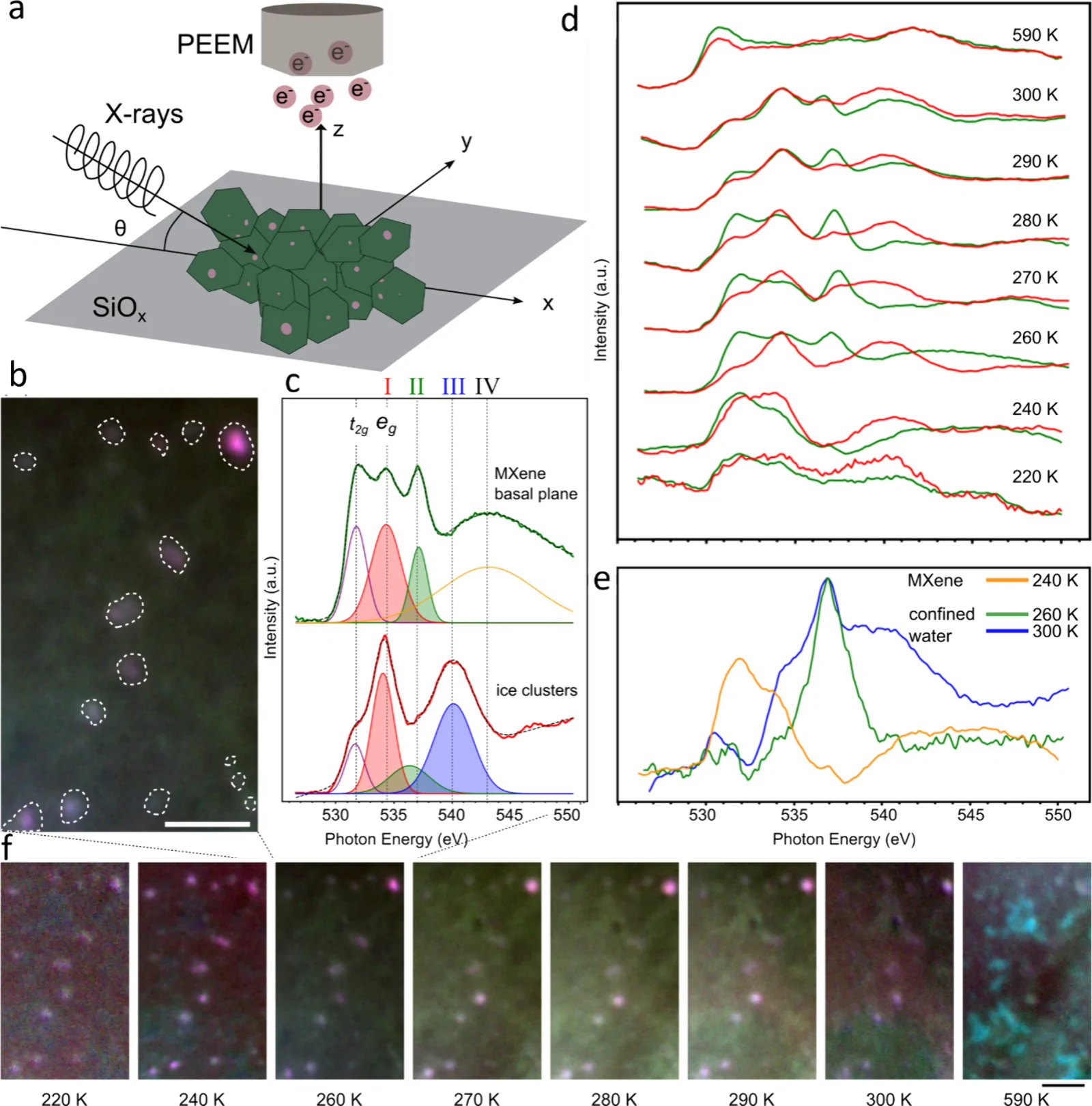
**Temperature-dependent XPEEM of Li–Ti**_**3**_ **C**_**2**_ **T*x* MXene.** a) Schematic of the *in situ* XPEEM imaging upon heating. b) XPEEM micrograph of a Li–Ti_3_C_2_T_*x*_ film at 260 K. The color scale is based on the respective contribution of the three water bands (I: red, II: green, III: blue) over the MXene film. Ice clusters are circled by dashed red lines. c) XAS at the O K-edge of the MXene film (green region) and ice clusters (purple region) are shown with their peak fitting. d) Temperature-dependent XAS at the O K-edge of the ice clusters (purple) and MXene film (black) between 220 and 590 K. The spectra are normalized and offset vertically for clarity. e) The XAS spectra from confined water are obtained by subtracting the XAS at 240 K from those recorded at 260 and 300 K. f) XPEEM micrographs over the 220 – 590 K temperature series. The scale bars are 2 μm. Reproduced with permission from reference.^3^ Copyright 2025 Springer Nature.

The characterization of confined water under a humid atmosphere has revealed a similar signature at the oxygen K-edge using *in situ* STXM.^2^ The electronic signature of the 2D confined water remains however different from bulk water, having three broad absorption bands corresponding to the various hydrogen bonding environments and resembling those of confined water in nanoporous materials, with sharper peaks.^57^ In humid air, gaseous (free) water was also identified close to the edges due to its intense and sharp absorption lines. Another type of hydrogen bonding was identified in water pockets composing the MXene film that is closer to bulk water based on its XAS spectrum. Note that other oxygen-containing molecules confined or adsorbed on the MXene surface, such as sulfates,^58^ can also be imaged thanks to their different chemical X-ray absorption that allows selective chemical imaging.

We have therefore identified different types of confined water within the MXene film in vacuum or in gaseous atmosphere. However, at this stage it remains unclear how these different hydrogen bonding are impacting the properties of the MXenes, but the presence of large pockets of bulk-like water probably alters the electronic conductivity of MXene films when immersed in water. Furthermore, the characterization in liquid water remains challenging due to the intense absorption of bulk water that screens intercalated species. In liquid, the probing of the transition metal within the MXene is more appropriate, as discussed in the next section.

## 6 Chemical Imaging of Intercalation Processes

The chemical imaging in an aqueous environment is facilitated at the Ti and V L-edges because their absorption energies lie below the O K-edge. As such, minimal absorption from the electrolyte is expected, which allows measurements in up to 10 μm-thick liquid films. As an example, few-layered Ti_3_C_2_T_*x*_ MXene flakes with three different thicknesses between 1 and 20 layers were imaged using STXM at the Ti L-edge under nitrogen flow (Figure 6a,b) and in various aqueous environments (Figure 6c,d).^2^ Short-term exposure to humid air does not significantly alter the MXene surface chemistry, but exposure to water and alkali cations (K^+^, Na^+^, or Li^+^) induces notable changes in Ti chemical bonding while the flake morphology remains intact (Figure 6e,f).

**Figure 6:**
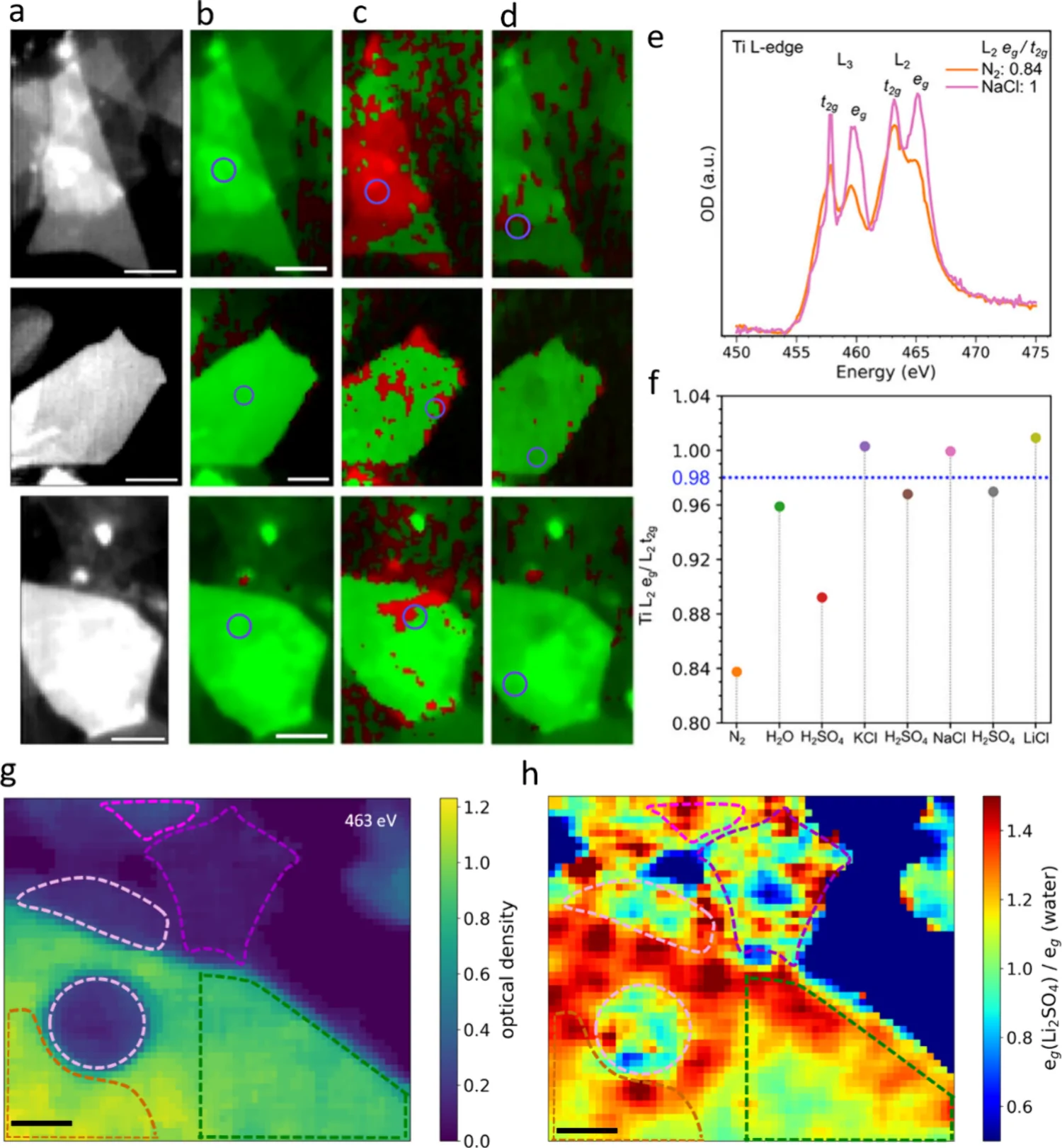
**Redox reaction on Ti**_**3**_ **C**_**2**_ **T_*x*_ MXene flakes in aqueous electrolytes.** a) STXM images of MXene flakes with thickness 1–10 layers (top), 10 layers (middle), and 20 layers (bottom), averaged over the Ti L-edge and exposed to N_2_ gas. STXM images at the Ti L-edge during exposure to b) N_2_ gas, c) H_2_O, d) 0.1 M H_2_SO_4_, colored based on L_2_ *e*_*g*_ to *t*_*2g*_ peak intensity ratio, reflecting the oxidation state of Ti atoms. Green (red) regions correspond to ratios lower (higher) than 0.98. e) XAS at the Ti L-edge for the 20-layered Ti_3_C_2_T_*x*_ MXene flake during exposure to N_2_ gas and 0.1 M NaCl aqueous electrolyte. f) XAS Ti L_2_ *e*_*g*_ to *t*_*2g*_ peak intensity ratio was evaluated for the 20-layered flake during sequential exposure to N_2_ gas, H_2_O, H_2_SO_4_, KCl, H_2_SO_4_, NaCl, H_2_SO_4,_ and LiCl (0.1 M for all electrolytes). The blue circles on the flakes indicate the area where the point scan XAS spectra were collected. Reproduced with permission from reference.^2^ Copyright 2026 Wiley. g) STXM image at the Ti L-edge (463 eV) in water. Highlighted regions correspond to thicknesses of 2 (purple dotted), 5 (pink dotted), 8 (light purple dotted), 30 (green dotted), and 35 layers (yellow dotted), respectively. h) Heat map of the intensity ratio of the L_2_ e_g_ peak in 0.1 M Li_2_SO_4_ and water (e_g_(Li_2_SO_4_)/e_g_(H_2_O)). Scale bars: 1 μm. Reproduced with permission from reference.^1^ Copyright 2026 Royal Society of Chemistry.

The XAS Ti L_2_ e_g_/t_2g_ peak intensity ratio was used to monitor the Ti oxidation state. A nearly pristine 20-layered flake under N_2_ shows a ratio of 0.84, which increases to 1 upon exposure to 0.1 M NaCl, indicating substantial surface oxidation. Chemical imaging using a threshold ratio of 0.98 highlights subflake regions with differing Ti oxidation states: thin flakes (1–10 layers) show widespread basal plane oxidation, whereas thicker flakes are oxidized primarily at the edges, which reversed on exposure to 0.1 M H_2_SO_4_ (Figure 6b,c,d). This pattern is attributed to easier water access in thin, more delaminated flakes and restricted interlayer penetration in thick flakes.

Sequential exposure to aqueous cationic solutions (0.1 M KCl, NaCl, LiCl) shows further Ti oxidation, partially reversible upon subsequent exposure to 0.1 M H_2_SO_4_ (Figure 6e,f). The reversible water- and cation-induced oxidation observed across the full area of the flakes confirms that redox-active sites are distributed over subflake regions, with hotspots related to surface defects or edges. X-ray beam-induced oxidation was observed near point-scan regions but did not alter the qualitative distribution of oxidation. However, this result highlighted that radiation damage in water cannot be ignored. In further work, we implemented fast raster scanning, for tracking spontaneous intercalation in 0.1 M Li_2_SO_4_ (Figure 6h).^1^ The L_2_ e_g_ ratio heat map between Li_2_SO_4_ and water (Figure 6g,h) enables a direct chemical imaging of the regions with oxidized Ti atoms at the flake level. This chemical oxidation is nonhomogeneous over the flakes, with a gradient from edge to basal plane. The e_g_ peak ratio heat map (Figure 6g,h) highlights regions of localized oxidation, showing approximately 93% active area on thick flakes (>9 layers) compared to 73% on thin flakes (≤9 layers). The lower fraction of active area in thin flakes further indicates that these flakes are more prone to water-induced oxidation.

Following spontaneous intercalation, the electrochemical H^+^ (de)­intercalation was monitored by applying an electrical potential *in situ* in a 3-electrode setup (Figure 7a). The cyclic voltammetry shows the characteristic pseudocapacitive response of Ti_3_C_2_T_*x*_ MXene with large reversible redox peaks centered around −0.5 V vs Pt (Figure 7a). The chemical imaging of the potential-induced change in MXene chemistry was performed on few-layered overlapping flakes with thicknesses ranging from 2 to 23 layers at the open circuit voltage (ocv, −0.13 V vs Pt) and applied −0.76 V (Figure 7b). The average Ti oxidation state depends on flake thickness. Bilayered flakes show negligible change under applied potential, whereas thicker flakes exhibit a subtle decrease in the L_2_ eg peak intensity at −0.76 V, corresponding to a Ti L_2_ e_g_/t_2g_ ratio drop from 0.81 to 0.77, indicating a Ti reduction of ∼ 0.1 e^–^/atom and confirming pseudocapacitive redox activity. The e_g_ peak ratio heat map calculated between −0.76 V and −0.13 V for the thick flakes (Figure 7c) shows an uneven distribution of the Ti oxidation state over the MXene flake. This corresponds to 64% of the active area in thin flakes and 88% in thick flakes. At this stage, the origin of the reduction of the active area remains unclear. For thin flakes, it could be due to water-induced oxidation. Local hotspots of higher Ti oxidation (inactive areas) are identified over the flake, which are more intense than the ones observed for spontaneous intercalation.

**Figure 7:**
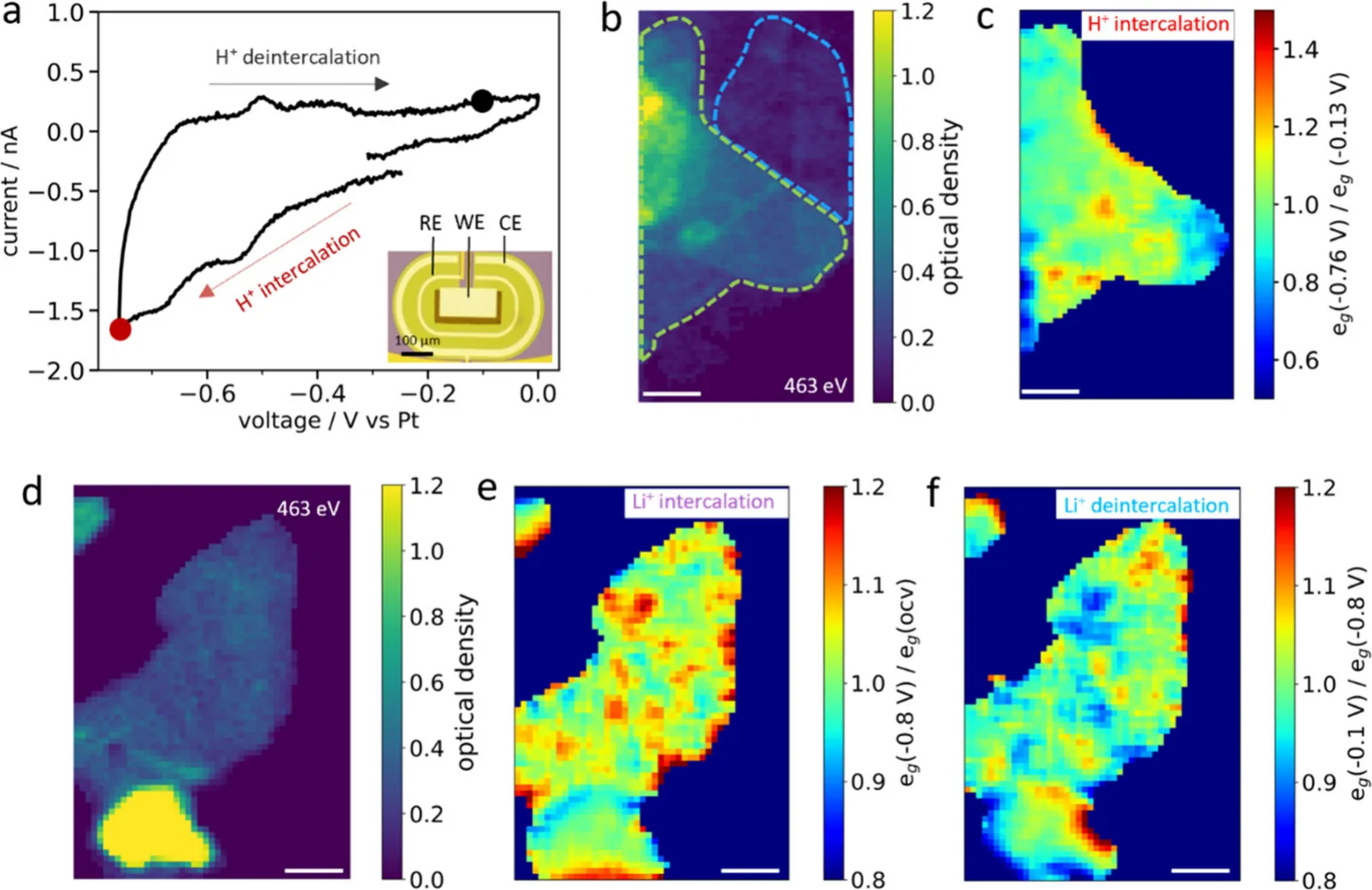
**Electrochemical ion intercalation in individual Ti**_**3**_ **C**_**2**_ **T_*x*_ MXene flakes.** (a) Cyclic voltammogram of Ti_3_C_2_T_*x*_ MXenes in 0.1 M H_2_SO_4_ measured using a 3-electrode electrochemical cell with a carbon working electrode and Pt counter and reference electrodes. Inset: optical image of an electrochemical chip showing the electrode geometry. (b) STXM image of Ti_3_C_2_T_*x*_ flakes measured at the Ti L-edge (463 eV) in 0.1 M H_2_SO_4_. The region of interest consists of thick, overlapping, few-layered MXene flakes (green) surrounded by bilayered MXene flakes (blue). (c) Heat map of the intensity ratio of the L_2_ e_g_ peak at −0.76 V and −0.13 V vs Pt (e_g_ (−0.76 V)/e_g_ (−0.13 V)). (d) STXM image at the Ti L-edge (463 eV) in 0.1 M Li_2_SO_4_ of ∼6- and 15-layered flakes. (e), (f) Heat map of the intensity ratio of the L_2_ e_g_ peak between −0.8 V vs Pt and the ocv (e_g_(−0.8 V)/e_g_(ocv)) for intercalation and between −0.1 V and −0.8 V (e_g_(−0.1 V)/e_g_(−0.8 V)) for deintercalation, respectively. Scale bars are 1 μm. Reproduced with permission from reference.^1^ Copyright 2026 Royal Society of Chemistry.

The electrochemical Li^+^ ion (de)­intercalation was further monitored in a 0.1 M Li_2_SO_4_ electrolyte on a few-layered Ti_3_C_2_T_*x*_ MXene flake (Figure 7d). Unlike H^+^ intercalation, the Ti atoms further oxidize upon electrochemical Li^+^ intercalation and reduce upon deintercalation. This counterintuitive behavior, opposite to the one observed in H_2_SO_4_, points to a more complex redox mechanism that does not involve the Ti atoms directly. The e_g_ ratio heat maps presented in Figure 7e and f show nonuniform oxidation and reduction across the flakes. Both thin and thick flakes exhibit approximately 70% electrochemically active area during oxidation, which decreases to around 60% during reduction. This decrease in active area likely results from irreversible oxidation caused by a combination of surface lithiation and water-induced oxidation. Similar to spontaneous intercalation, thinner flakes exhibit localized “hotspots” of activity across the basal plane. The origin of this subflake inhomogeneity remains unclear. It may be associated with surface defects or surface chemistry, although the size of the clusters (few 100s nanometers) extends much beyond what may be expected for defective regions. Connecting to the previous results on confined water, it may be that pockets of water with a different hydrogen bonding due to buckling of the MXene flake may affect the local electrochemical activity of the MXene flake. In contrast, thicker flakes show activity primarily along their edges, where defects are more likely to be present. Further work is needed in this direction to clarify the nature of local MXene heterogeneity in their electrochemical activity.

Overall, these *in situ* STXM measurements have brought a new understanding of intercalation processes in MXenes. Especially, we showed that cation intercalation does lead to charge transfer to the MXene in the first cycles, while only adsorption of ions in the double layer was initially expected. We also revealed that the MXene electrochemical activity is not uniform over the flakes, which suggests that there is still room for improvement for their performance by controlling the morphology of the flakes or their surface chemistry. Of course, questions remain about how these results obtained on single flakes may be extrapolated to thick electrodes, which may present quite different morphologies and induce additional transport processes.

## 7 Future Directions of Chemical Imaging of 2D Materials

As demonstrated in this account, chemical imaging with X-ray microscopy is a powerful technique for understanding local chemical processes occurring at the nanoscale and identifying intercalated species. First *in situ* studies were performed on intercalation processes in MXenes but many other chemical reactions may probably be imaged. Hydrolysis reactions leading to degradation of MXene flakes may be studied, as may electrocatalytic reactions such as the hydrogen evolution reaction (HER), although the formation of bubbles is challenging experimentally.^59^

X-ray chemical imaging can also be applied to other 2D materials, such as graphene oxide^34^ and layered oxides.^30^ Beyond Ti_3_C_2_T_*x*_, V-, Nb and Mo-based MXenes may also be investigated by soft X-ray microscopy. This may be of interest as V_2_CT_*x*_ have demonstrated outstanding properties for electrochemical energy storage, especially in Zn-ion batteries,^60^ while Mo_2_CT_*x*_ are particularly promising for HER.^61^ The high chemical and spatial resolution is also well adapted to probe composite materials and 2D heterostructures. Coming back to LFP, current *in situ* X-ray chemical imaging platforms could also be operated in organic electrolytes, which are more standard for industrial applications. Indeed, while measuring Ti_3_C_2_T_*x*_ flakes in an organic solvent can be very challenging since the Ti L-edge is only 150 eV above the C K-edge and the X-rays are therefore strongly attenuated (ca. 30% transmission in the case of a 1 μm thick layer of ethylene carbonate), the attenuation at the Fe L-edge is roughly the same whether the solvent is water or ethylene carbonate. Achieving *in situ* measurements in organic solvents does not seem to represent a major conceptual challenge, but could open new avenues for the use of STXM in the battery field.

While X-ray microscopy offers high versatility, it may be beneficial to correlate it with other microscopy techniques. For example, correlative electron and X-ray microscopy are promising to benefit from the higher spatial resolution of electron microscopy with the chemical contrast of X-ray microscopy.^62^ The combination with scanning electrochemical probe microscopy also seems of particular interest because it would allow the correlation of local chemistry with the chemical structure, as recently demonstrated on transition metal oxyhydroxide.^30^ The use of nitrogen-doped thin carbon support are particularly well adapted for such correlative microscopy approaches.^58^

Finally, novel imaging techniques are currently emerging that may allow a higher spatial resolution or extend to 3D imaging. These techniques will particularly benefit from the latest upgrades in modern synchrotron facilities, which ensure higher brilliance and coherence. X-ray spectro-ptychography uses the diffraction of coherent light to achieve similar chemical information as STXM but without being diffraction-limited in terms of spatial resolution,^63^ although its application to *in situ* electrochemistry remains scarce. It would be particularly beneficial for *in situ* studies because of the reduced sample damage ensured by the use of a defocused X-ray beam.

The combination of X-ray chemical sensitivity with laminography may also provide new avenues for the 3D chemical imaging of MXene flakes at the nanoscale.^64^ In particular, it was recently reported that multilayered flakes present higher conductivity than monolayered flakes,^65^ which suggests different surface chemistry between their top and inner layers. 3D X-ray imaging may be able to resolve chemistry inhomogeneities in few-layered MXene flakes and related materials. Nevertheless, sample rotation during tomography substantially increases the effective path length, resulting in low transmission that may become an issue for soft X-ray imaging. Extending X-ray microscopy to the hard X-ray region, measuring at the transition metal K-edge, would also be of high interest to increase the penetration depth of the technique and be able to characterize full multilayered MXene flakes or even films, which are closer to commercial systems.

## 8 Conclusions

X-ray chemical imaging allows the characterization of chemical processes occurring at the subflake level on individual MXene flakes, down to monolayers. We have shown in our recent work that this method offers a large sensitivity to local chemical changes. The element and site specificities allow to selectively excite atoms from intercalated water and follow change in their hydrogen bonding with spatial resolution. Transition metal atoms in the MXene plane can also be excited to probe local metal bonding, which can be tracked directly in liquid and/or under application of an electrochemical potential. This has been highlighted by considering the intercalation processes of protons and Li^+^ cations in the interlayer spacing of the Ti_3_C_2_T_*x*_ MXene flakes. While most of published work are currently focusing on Ti_3_C_2_T_*x*_ MXene as discussed in this account, other MXenes are certainly worth consideration. We are, however, still at the beginning of this field and there is certainly room for new discoveries thanks to X-ray chemical imaging in the coming years.
